# Hürthle Cell Carcinoma: Single Center Analysis and Considerations for Surgical Management Based on the Recent Literature

**DOI:** 10.3389/fendo.2022.904986

**Published:** 2022-06-29

**Authors:** Costanza Chiapponi, Milan J.M. Hartmann, Matthias Schmidt, Michael Faust, Christiane J. Bruns, Anne M. Schultheis, Hakan Alakus

**Affiliations:** ^1^ Department of General, Visceral, Cancer and Transplant Surgery, University Hospital Cologne, Cologne, Germany; ^2^ Department of Nuclear Medicine, Faculty of Medicine, University Hospital Cologne and University Hospital Cologne, Cologne, Germany; ^3^ Polyclinic for Endocrinology, Diabetes and Preventive Medicine, University Hospital Cologne, Cologne, Germany; ^4^ Department of Pathology, University Hospital Cologne, Cologne, Germany

**Keywords:** Hürthle cell carcinoma, thyroid cancer, prophylactic lymphadenectomy, lymph node involvement, recurrence of HCC

## Abstract

**Background:**

Hürthle cell carcinoma (HCC) of the thyroid is rare. There are contrasting data on its clinical behavior. The aim of this study was to describe clinic-pathological features and outcomes of HCC patients at our institution, in order to adapt our surgical management.

**Methods:**

We retrospectively studied 51 cases of HCC treated at the interdisciplinary endocrine center of the University Hospital of Cologne, Germany between 2005 and 2020.

**Results:**

Patients median age was 63 years (range 29-78) with 64.7% of cases being female. Primary treatment included surgery and postoperative radioiodine therapy with 3.7 GBq in all patients. Surgery consisted of total thyroidectomy in all cases and additional central lymphadenectomy in 90.2% of cases. The median number of harvested lymph nodes was 11 (range 2-31). Lymph node involvement was found in two (4.3%) pT4a tumors. In all other cases (95.7%), central lymphadenectomy was prophylactic and lymph nodes were free of metastasis in final histopathology. Twelve (23.5%) patients with incomplete biochemical response to primary treatment were diagnosed with structural relapse during the course of disease, for which seven (58.4%) underwent resection of isolated cervical metastasis. Histopathology revealed soft tissue implants in all cases and cervical surgery led to biochemical and radiologic cure in only two (28.5%) cases. Five (41.6%) patients developed metastatic disease, followed by systemic therapy in two patients. Vascular invasion of the primary tumor was significantly associated with relapse (p<0.01).

**Conclusions:**

Recurrence of HCC was common in this study. Given the low rate of lymph node metastases both in this study and in recent literature and the nature of relapse (soft tissue instead of nodal metastasis), the benefit of routine prophylactic central lymph node dissection for HCC remains unclear, especially in the absence of vascular invasion from the primary tumor.

## Introduction

Hürthle cell carcinoma (HCC) of the thyroid is rare. It is reported to account for only 3-4% of all thyroid tumors (1). A recent retrospective analysis of HCC primarily treated with surgery at Memorial Sloan Kettering Cancer Center between 1986 and 2015 identified a total of 111 patients only (2).

HCC was previously classified as a variant of follicular thyroid carcinoma but was reclassified in 2017 in the 4^th^ WHO classification of Tumors of Endocrine Organs as a separate subtype of differentiated thyroid cancer (3), due to its distinct genetic expression profile, pathologic characteristics, and clinical behavior. Higher frequencies of distant metastasis, impaired iodine-avidity, and higher rates of mortality have been reported (4, 5), but data are controversial (6–8). Recently, Jin et al. (1) reported persistent/recurrent disease in only 8.2% of the patients, and none of their patients died of HCC during a median follow-up of 8.5 years. Similarly, Oluic et al. (9) reported a rate of 12% of persistence/recurrence, with a slightly worse survival rate than Jin et al.

According to the German Guidelines (10), a routine prophylactic central lymph node dissection is recommended in case of preoperative or intraoperative HCC diagnosis. This recommendation is explained by high rates of lymph nodal involvement and impaired iodine avidity of HCC. In case of postoperative diagnosis, prophylactic central lymphadenectomy is recommended for biochemical residual disease, consisting in elevated thyroglobulin level (Tg). These recommendations are based on seven studies (11–17): two reviews (12, 16) and four retrospective analyses (11, 13, 15, 17). They include Lopez et al. (11) reporting a 25% lymph node involvement and a 40% mortality rate in 89 patients, Kushchayeva et al. (13) with 3% lymph node metastasis at presentation and 13% in follow up in 33 patients and Guerrero et al. (17) with lymph node involvement in only 3 (8%) of their 39 patients and in no tumor <5cm. The fourth study quoted in the guidelines is Besic et al. (15). It describes 30.9% of lymph node involvement and 45% of extrathyroidal extension in 42 patients. However, these patients were diagnosed with papillary Hürthle cell carcinoma, which is a variant of papillary thyroid cancer.

Given the conflicting data in the literature, we aimed at investigating clinic-pathological features and outcomes of HCC patients treated at our institution, in order to critically evaluate surgical management of these patients.

## Methods

### Patients and Tumors

Patients, who underwent radioiodine treatment (RAIT) between 01.01.2005 and 31.07.2020 at the University Hospital of Cologne, Germany for differentiated thyroid cancer, were screened. The group of Hürthle cell carcinomas (HCC) according to the current WHO definition was identified [more than 75% of the tumor consists of Hürthle cells (18)]. Papillary Hürthle cell carcinoma is a distinct entity and was not included in the present study. Patients receiving surgical treatment at our institution as well as those referred to our center for RAIT and oncologic care after external surgery were included in the present study.

Histologic classification was made according to the at the time current IACR WHO Classification of Tumors of Endocrine Organs, representing Hürthle cell carcinoma in the current 2017, 4^th^ edition of the WHO Classification of Tumors of Endocrine Organs. Staging was performed using the UICC TNM classification of malignant tumors (18).

### Primary Treatment

According to the current German Guidelines (10), primary surgical management for pre- or intraoperatively diagnosed HCC consists of total thyroidectomy and prophylactic central lymphadenectomy. For postoperatively diagnosed HCC two-stage thyroidectomy is recommended. For patients with still elevated thyroglobulin levels or suspicious lymph nodes after total thyroidectomy, lymphadenectomy is suggested (10).

RAIT is recommended for all patients. At our institution diagnostic whole-body scintigraphy scan (DWBS) is regularly performed 6-9 months after RAIT and represents the only staging parameter routinely performed for differentiated thyroid cancer, besides comprehensive cervical sonography. SPECT/CT of the neck and chest completes the staging.

### Diagnosis and Radioiodine Treatment for Recurrent Disease

Diagnosis of recurrence was based on physical examination, thyroglobulin (Tg) increment and imaging studies.

During follow up, significant elevation of basal and stimulated serum Tg compared to the nadir value as well as all values >1 ng/mL measured with an ultrasensitive assay led to a diagnostic whole-body scintigraphy scan (DWBS) with 185-370 MBq of radioiodine, followed by ^18^F-FDG PET-CT if radioiodine uptake was low or absent. If DWBS studies were positive, patients received a therapeutic activity of 3.7 of radioiodine (I-131). If DWBS was negative, but ^18^F-FDG PET-CT confirmed structural recurrence, surgery was considered. If the imaging studies did not display recurrence, serum TG levels were monitored, as described previously (19).

### Surgery of Recurrence

Indication to surgery of recurrence in our institution is always initiated by the multidisciplinary endocrine team (MDET). Surgery consists in resection of isolated soft tissue tumors in the perithyroidal/paratracheal space, in case structural recurrence does not appear iodine avid in DWBS or is deemed too large for repeated radioiodine therapy. Surgery is always performed by a specialized endocrine surgeon with intraoperative frozen section and neuromonitoring (19).

### Follow-Up

Follow-up examinations take place every 6 months for 5 years and thereafter yearly in the department for Nuclear Medicine and include physical examinations, cervical ultrasound and laboratory values including thyroglobulin. DWBS, MRI and/or ^18^FDG PET-CT are planned if necessary. Follow up examinations until March 2022 were included in this study. Mean follow up was 62 ± 55.9 months after thyroidectomy. Response was evaluated according to the RECIST criteria.

### Data Collection and Analysis

Electronic and paper data of the University Hospital of Cologne were retrospectively collected and analyzed. The study was performed according to the rules and regulations for retrospective analysis of the ethical committee of the University Hospital Cologne. Data were analyzed using IBM SPSS Statistics for Windows, Version 25.0. Armonk, NY. Variables were expressed as median with range. If the data was normally distributed, groups were compared using the T-test. The Chi-square test of independence was used for testing hypotheses when the variables were nominal. A p-value < 0.05 was considered significant.

## Results

### Patient Characteristics

Among 1897 patients receiving radioiodine treatment (RAIT) for differentiated thyroid cancer, 51 (2.7%) were diagnosed with HCC (**Table 1**). Their median age was 63 years (range 29-78). Eighteen (35.3%) patients were male and had a median age of 57 (range 29-76). 33 (64.7%) patients were female and were in median 64 (range 29-78) years old. There was no significant gender specific difference in their age (p=0.27).

**Table 1:** Demographic and tumor characteristics of HCC patients included in the present study.

N= 51	
**Age (median, range)**	63 (29-78)
**Gender**	
Male Female	18 (35.3%) 33 (64.7%)
**pT status**	
pT1 pT2 pT3 pT4	10 (19.6%) 26 (51%) 11 (21.6%) 4 (7.8%)
**pN status**	
pN0 pN1 pNx	44 (86.2%) 2 (3.9%) 5 (9.8%)
**pM status**	
pM1	2 (3.9%)
**V status**	
**V0** **V1/2** **Vx**	21 (41.2%) 21 (41.2%) 9 (17.6%)

### Tumor Stages at the Time of HCC Diagnosis

Ten (19.6%) patients had pT1b tumors, 26 (51%) had pT2, 11 (21.6%) had pT3 and 4 (7.8%) had pT4 status. Central lymphadenectomy was performed in 46 (90.2%) patients and delivered a median of 11 lymph nodes (range 2-31). In 2 (4.3%) of 46 cases lymph node metastases were suspected on preoperative radiologic examination and confirmed in final pathologic exam. Both patients had pT4a status. Distant metastases were present in 2 (3.9%) patients at diagnosis, both with pT4a tumors. One of these two patients had no lymph node involvement.

V-status was documented in 42 (82.3%) pathology reports and was positive (V1/V2) in 21 (50%) cases, including 3 (30%) of 10 pT1, 9 (34.6%) of 26 pT2, 6 (54.5%) of 11 pT3 and 3 (75%) of 4 pT4 tumors.

### Radioiodine Treatment

All patients received at least one course of radioiodine therapy. In forty-one (80.4%) cases only one course with an activity of 3.7 GBq ^131^I was administered. In four (7.8%) patients a higher initial dose (5.5 GBq) and in six (11.7%) patients more than one radioiodine treatment were administered during the course of disease, with a median cumulative dose of 7.4 GBq (range 5-55 GBq) ^131^I.

### Repeated Cervical Surgery

Second-line surgery for isolated cervical relapse was required in 7 (13.7%) of 51 patients. 71.4% of these patients were male. Their primary tumors consisted of 4 (57.1%) pT2 and 3 (42.9%) pT3 status with vascular invasion (V1/2) in 5 (83.4%) of six pathology reports with V-status documentation. None of them had radiologic (28.5% cN0) or pathologic (71.4% pN0) lymph node involvement at the time of diagnosis. In all cases, initial postoperative radioiodine treatment had resulted in an incomplete biochemical response. **Table 2** summarizes the clinic pathological features and outcome of these cases.

**Table 2:** Clinic-pathological features of patients undergoing second-line surgery for isolated cervical relapse without distant metastasis.

Pt number	Gender, Age	pT	pN	cM	V	Cumulative RAI activity (GBq)	Follow up (months)	End result (response) • excellent = ER • indeterminate = IR • biochemical incomplete = BI • structural incomplete = SI
**1.**	**M, 56**	pT2	Nx	0	1	3.7	135	ER
**2.**	**M, 74**	pT3a	0/17	0	0	3.7	59	BI
**3.**	**M, 56**	pT3a	0/2	0	1	3.7	42	IR
**4.**	**M, 38**	pT3a	0/7	0	2	3.7	36	BI
**5.**	**F, 59**	pT2	0/7	0	1	5.5	211	IR
**6.**	**F, 72**	pT2	Nx	0	1	7.4	174	ER
**7.**	**M, 76**	pT2	0/10	0	1	3.7	68	SI

Table 2 summarizes the clinic-pathological features of patients undergoing second-line surgery for isolated cervical relapse without distant metastasis. Tg, Thyroglobulin. None of these patients had pathologic Tg antibodies. V= angioinvasions (V0= absent, V1= microscopic, V2= macroscopic). End results were classified in excellent, indeterminate, biochemical incomplete and structural incomplete response according to Pitoia and Jerkovich (20).

**Figure 1:**
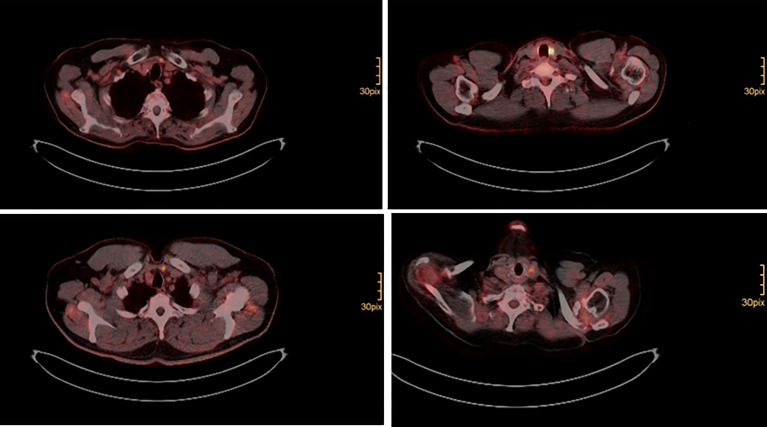
Isolated cervical FDG positive relapse in four patients removed by surgery.

**Figure 2:**
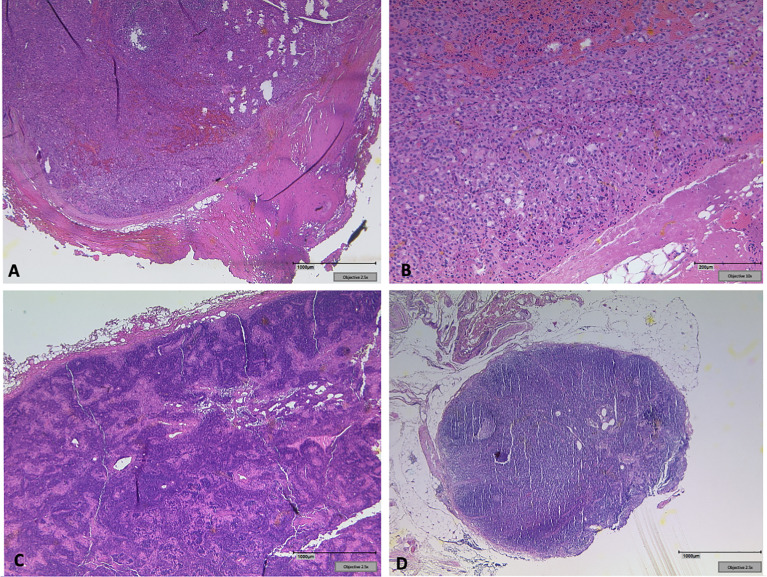
**(A)** Soft tissue implant showing a well-circumscribed tumor nodule partially surrounded by a fibrous capsule clinically mimicking lymph node metastasis. **(B)** Oncocytic tumor cells form small follicles, sharply separated from surrounding soft tissue. No lymphatic tissue detectable. **(C)** Tumor free lymph node next to the left jugular vein. **(D)** Exemplary clear lymph node from the central compartment in the same patient.

Surgery was performed, when structural radioiodine refractory disease was diagnosed during the course of disease (**Figure 1**). In all cases final pathology of relapse revealed soft tissue metastases: three paratracheal implants in the central compartment, three jugular tumors in the lateral compartment, one supraclavicular tumor ventral of the sternocleidomastoid muscle. There were no metastatic lymph nodes. **Figure 2** shows how a well circumscribed tumor nodule partially surrounded by a fibrous capsule can mimic lymph node metastasis in radiologic studies (**Figure 2**).

### Overall Outcome

After primary treatment, an excellent response was recorded in 38 (72.5%) patients. One (1.96%) patient had an indeterminate response. In 12 (70.6%) patients with initial incomplete biochemical response, structural recurrence was diagnosed during the course of disease.

Seven (58.3%) patients with structural recurrence displayed isolated cervical tumors, which were removed surgically (**Table 1**). Two of these patients displayed an excellent response at last follow up (28.6%). Five (71.4%) presented an indeterminate (n=2), biochemical incomplete (n=2) or structural incomplete response at last follow up (n=1, stable lung metastases in a follow up of 68 months).

Five (41.7%) patients with structural persistence/recurrence were diagnosed (n=2, 40%) or developed (n=3, 60%) distant metastases in the course of disease and did not receive a second cervical procedure after primary thyroidectomy. Their metastases consisted of pulmonary and/or bone lesions. These patients were older than 65 years (median age 69, range 29-77), with exception of one 29 year old female patient. They were offered tyrosine kinase inhibitors as a systemic treatment but refused in three cases. In both patients receiving systemic treatment, therapy included lenvatinib in first line, followed by nivolumab and pembrolizumab, due to side effects of lenvatinib. Both patients discontinued systemic treatment and died two and four years after thyroidectomy respectively. Both patients had widely invasive pT4a tumors and were 66 and 69 years old at the time of diagnosis. The other three experienced slow disease progression.

Recurrence was significantly more common in tumors, in which vascular invasion (V1 and 2) had been documented (p<0.01).

## Discussion

In the present study, the clinic-pathologic features and clinical course of 51 patients treated at our institution in a period of 15 years were analyzed. Despite initial surgery and radioiodine treatment, recurrence rate was as high as 23.5%, which is similar to that previously reported in our patients with follicular thyroid cancer (21) but higher than that reported in some recent literature (1, 2). The reasons underlying these differences are unclear and might be of demographic, ethnic or environmental nature.

There is an increasing number of recent studies questioning the reported aggressiveness of HCC. Jin et al. (1) reported persistence/recurrence rates of 8% and no death in a follow up of 8.5 years in South Korea, a region with a good iodine intake. Matsuura et al. (2) compared HCC to FTC in a US American population and found that HCC patients were more likely to recur, but they had shown no significant different overall survival. Vascular invasion was a critical prognostic parameter for predicting recurrence in their study (2). The current German Guidelines recommend total thyroidectomy and routine prophylactic central lymphadenectomy for Hürthle cell carcinoma irrespective of V status (10).

Due to the impaired iodine avidity of HCC (22) and the assumed high rate of lymph node involvement, the guidelines recommend prophylactic central lymphadenectomy (10). Accordingly, this was regularly performed (90.2% of cases). However, despite a sufficient median number of harvested lymph nodes (n=11), lymph node metastases were found in only two (4.3%) patients presenting with pT4a status. This rate is similar to that of Goffredo et al. (23), who documented node-positive disease in 5.3% of cases. The only two studies reporting a high nodal involvement are Chindris et al. (24) (21.9%) and Lopez et al. (11). However, it needs to be mentioned that in Chindris et al. lymph nodes were not examined in the majority of cases (57.8%), therefore the rate of lymph node involvement is probably not representative. The collective of Lopez et al. (11) includes 7 tumors with anaplastic foci, which might influence nodal involvement rates.

Salvage surgery for loco regional relapse can deliver information on the nature of recurrence. In this study, it was performed in 7 (13.7%) patients and revealed soft tissue metastases instead of lymph node metastases (**Figure 1**). This result is consistent with Bishop et al. (25), who analyzed 24 metastases and reported that there were no true lymphatic metastases in the majority of cases, but tumor emboli in veins that reimplant along the venous outflow tract. These metastatic nodules were not surrounded by any peripheral cuff of lymphoid tissue or capsule in most cases (25). This important aspect is probably not given enough clinical attention. These data together with those of Coca Pelaz et al. (26), suggest that prophylactic central lymphadenectomy might not be of benefit in HCC. In their meta-analysis Coca Pelaz et al. included 9638 patients and found lymph node metastasis in only 9% of patients (26).

Soft tissue metastatic nodules can generally be identified in preoperative ^18^F-FDG PET scan (**Figure 1**). However, surgeons should be aware of the presence of additional sub centimeter foci, which might sometimes be found only by accurate palpation and intraoperative sonography and sometimes are even missed. In fact, only 2 (28.6%) of the patients undergoing cervical salvage surgery in the present analysis could be cured. All others had at least biochemical residual disease (**Table 2**). A recent retrospective analysis of the National Cancer Database (NCDB) suggested improved survival for HCC patients, who receive radioiodine treatment (27). Whether adjuvant external beam radiation therapy (EBRT) might be useful in these patients is not fully clear to date (28). There is a lack of evidence on EBRT for thyroid cancer recurrence.

Five (9.8%) patients with progressive metastatic disease were not referred to surgery in this study. Two of these patients agreed to tyrosine kinase treatment but treatment had to be changed or discontinued due to side effects. Both patients died two and four years after diagnosis respectively. A particular challenge for systemic treatment is the general lack of actionable somatic alterations in HCC (29). Alterations amenable to targeted therapy seem to be rare (30, 31).

It needs to be mentioned that not all pathologic specimens were available for review. In some cases, only external pathology reports were used for diagnosis and treatment. Despite a new WHO classification of Hürthle cell tumors though, 75% Hürthle cells in specimen was also required before 2017 for Hürthle cell diagnosis. It also needs to be mentioned the high persistence/recurrence rate reported (23.5%), might be even higher, given an average time to relapse of 90.7 months, as reported in some studies (2, 32, 33). Finally, due to the rarity of HCC, the collective included is not large enough for formulating general recommendations. However, the data presented, and especially the rate of lymph node involvement are similar to those in the meta-analysis of Coca Pelaz et al. (26).

Since most of the patients in this study underwent a central lymphadenectomy, one might speculate on whether this may have contributed to the absence of lymph nodes in the loco-regional recurrences. However, the data of Coca Pelaz et al. (26) and those of Bishop et al. (25) also suggest that lymph nodal involvement is a rather rare event and most recurrences consist of soft tissue implants. Based on the present data, on the literature and on our clinical experience, it appears that prophylactic central lymphadenectomy does not avoid recurrence, which mostly consists of soft tissue metastasis, thus suggesting hematogenous rather than lymphogenous spread. Current evidence does not appear sufficient for recommending routine central lymph node dissection for HCC, especially in the absence of vascular invasion.

## Data Availability Statement

The raw data supporting the conclusions of this article will be made available by the authors, without undue reservation.

## Ethics Statement

The studies involving human participants were reviewed and approved by Ethics board of the University of Cologne 50931 Cologne. Written informed consent for participation was not required for this study in accordance with the national legislation and the institutional requirements.

## Author Contributions

Conceptualization, CC, HA, and MS; methodology and software CC; formal analysis, HA, MF, MS, MH, CB and AS; investigation, CC and MH; resources, MH, MS; data curation, CC; writing—original draft preparation CC, HA and MH; writing—review and editing, MS, MF, AS, and CB. All authors have read and agreed to the published version of the manuscript.

## Conflict of Interest

The authors declare that the research was conducted in the absence of any commercial or financial relationships that could be construed as a potential conflict of interest.

## Publisher’s Note

All claims expressed in this article are solely those of the authors and do not necessarily represent those of their affiliated organizations, or those of the publisher, the editors and the reviewers. Any product that may be evaluated in this article, or claim that may be made by its manufacturer, is not guaranteed or endorsed by the publisher.
